# Mechanical Properties and Durability of Ultra High Strength Concrete Incorporating Multi-Walled Carbon Nanotubes

**DOI:** 10.3390/ma9060419

**Published:** 2016-05-27

**Authors:** Liulei Lu, Dong Ouyang, Weiting Xu

**Affiliations:** 1 Department of Mechanics and Civil Engineering, Jinan University, Guangzhou 510632, China; luliulei521@163.com; 2 Research Center of Engineering Materials and Structural Durability, Jinan University, Guangzhou 510632, China; weitinxu@gmail.com

**Keywords:** carbon nanotubes, ultra high strength concrete (UHSC), compressive strength, chloride permeability

## Abstract

In this work, the effect of the addition of multi-walled carbon nanotubes (MWCNTs) on the mechanical properties and durability of ultra high strength concrete (UHSC) is reported. First, the MWCNTs were dispersed by a nano sand-mill in the presence of a surfactant in water. The UHSC specimens were prepared with various amounts of MWCNTs, ranging from 0% to 0.15% by weight of cement (bwoc). Results indicated that use of an optimal percentage of MWCNTs (0.05% bwoc) caused a 4.63% increase in compressive strength and a 24.0% decrease in chloride diffusion coefficient of UHSC at 28 days curing. Moreover, the addition of MWCNTs also improved the flexural strength and deformation ability. Furthermore, a field-emission scanning electron microscopy (FE-SEM) was used to observe the dispersion of MWCNTs in the cement matrix and morphology of the hardened cement paste containing MWCNTs. FE-SEM observation revealed that MWCNTs were well dispersed in the matrix and no agglomerate was found and the reinforcing effect of MWCNTs on UHSC was thought to be pulling out and microcrack bridging of MWCNTs, which transferred the load in tension.

## 1. Introduction

Concrete, a quasi-brittle construction material commonly used in the world, has a relatively high compressive strength, but low flexural and tensile strengths [1]. Moreover, cracks are one of the main hidden defects in concrete structures; they cause brittle fracture, shorten the service life, and lower the durability [2,3,4,5]. Generally, the damage and failure of concrete are caused by the nucleation, growth, and coalescence of microcracks [6,7,8]. One strategy to inhibit the formation of cracks is to randomly introduce short discrete fibers into concrete [9]. After the microcracks coalesce into macrocracks, traditional microfibers (e.g., steel fiber or polypropylene fiber) mitigate their unstable propagation by providing effective bridging, strength, toughness, and ductility [9,10,11,12,13,14]. However, these microfibers cannot stop or prevent the initiation of microcracks in a concrete matrix [15,16].

In the past decade, carbon nanotubes (CNTs), discovered by Iijima in 1991 [17], have attracted much interest among cement and concrete researchers. CNTs are quasi one-dimensional carbon nanomaterials with aspect ratios ranging from 30 to more than many thousands, and they can be classified as single-walled CNTs (SWCNTs) rolled up by one-layer graphene sheet and multi-walled CNTs (MWCNTs) rolled up by multilayer graphene sheets [18,19]. CNTs have excellent mechanical properties, with elastic modulus of approximately 1 TPa, tensile strength of over 50 GPa and yield strain of up to 10%–20% [20,21,22]. These unique properties of CNTs may be help to prevent the initiation of microcracks in cement matrix at very early times [1,19]. Therefore, CNTs can be used as reinforcement materials of cement composites. Previous studies showed that the addition of CNTs (especially MWCNTs) improved the mechanical properties [15,16,23,24,25,26,27,28,29,30] and durability [31,32,33] of cement composites.

Li *et al.* [23] reported that the addition of 0.5% surface-treated MWCNTs by weight of cement (bwoc) into cement mortar increased the compressive and flexural strengths by 19% and 25%, respectively. Yakovlev *et al.* [24] reported that the use of CNTs (0.05% bwoc) increased the compressive strength of a cement-based foam concrete by 70%. Konsta-Gdoutous *et al.* [25] reported that the incorporation of MWCNTs (0.08% bwoc) to cement paste caused a 35% increase in flexural strength. Sobolkina *et al.* [28] reported that the modification of a cement paste with CNT dispersions significantly increased the compressive strength and moderately increased the tensile strength under a high strain rate loading. Hu *et al.* [15] reported that the addition of MWCNTs with a carboxyl group (0.1% bwoc) into cement paste improved the fracture energy and fracture toughness by 42.9% and 19.2%, respectively. Zou *et al.* [30] reported that the use of MWCNTs significantly improved the elasticity modulus, flexural strength, and fracture energy of cement pastes. Yang *et al.* [32] reported that the addition of MWCNTs increased the impermeability and abrasion resistance of cement composites. The addition of MWCNTs also efficiently improved the resistance to chloride ion penetration and sulfate attack [33]. Moreover, the addition of MWCNTs improved the microstructure of cement matrix, such as it decreased the porosity and increased microcrack bridging capacity at a nanoscale level [23,30]. This helped to improve the mechanical and durability properties of cement composites. Furthermore, the addition of MWCNTs with high electrical property increased the pressure-sensitive properties of cement composites [34,35].

Because of the low yield and high cost of MWCNTs, previous studies have focused on the effect of MWCNTs on the performance of cement pastes or mortars; however, the properties of concrete, especially ultra high strength concrete (UHSC) have been rarely reported. Modern high-rise buildings are built higher and larger and in ever increasing numbers, and the application of UHSC has become an inevitable trend. The compressive strength of UHSC is >100 MPa, but its safety has been questioned because of possible ultra-brittle failure behavior [36,37].

In the present study, the effect of MWCNTs on the mechanical properties and durability of UHSC was investigated. First, the MWCNTs were dispersed by a nano sand-mill in the presence of a surfactant (noncovalent treatment) in water. Moreover, the dispersion of MWCNTs in the cement matrix and morphology of the hardened cement paste containing MWCNTs (0.3% bwoc) were observed using a field-emission scanning electron microscope (FE-SEM).

## 2. Experimental Section

### 2.1. Materials

The properties of MWCNTs are shown in Table 1. Polyvinyl pyrrolidone (PVP), a commercially available surfactant, was used for dispersing MWCNTs in water. Ordinary Portland cement type II 42.5R (C), silica fume (SF), and ground granulated blast-furnace slag (BS) were used in all mixtures. The chemical compositions and physical properties of C, SF, and BS are listed in Table 2. A polycarboxylate-based superplasticizer (SP) was used in concrete mixtures for workability purposes. The fine aggregate (FA) used in this study was natural river sand with a fineness modulus of 2.79. The coarse aggregate (CA) was crushed granite with a maximum size of 20 mm. Moreover, tributyl phosphate (TP) was used for defoaming.

### 2.2. Dispersion of MWCNTs

**Figure 1:**
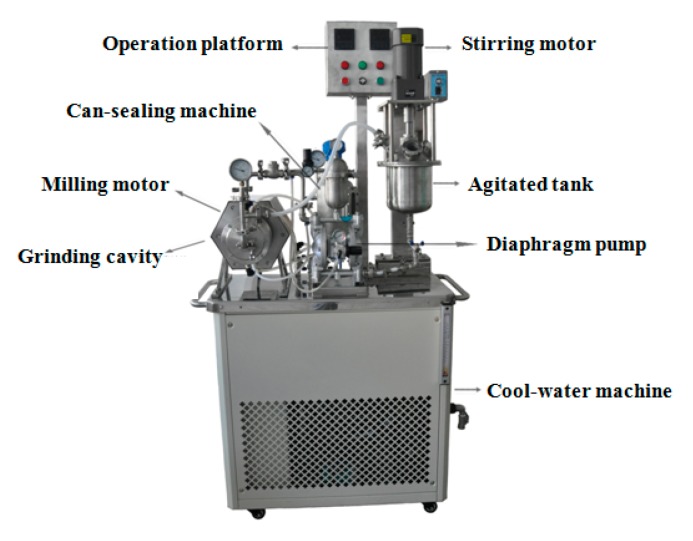
Image of the nano sand-mill.

The MWCNTs were dispersed using PVP by the surfactant-milling method. The mass ratio of the MWCNTs to the dispersant was 1:2, and the dispersant was first dissolved in water. Then, the weighed MWCNTs were added into the aqueous dispersant solutions and well dispersed at room temperature for 2 h using a BYZr-0.3 L turbo-type nano sand-mill (Figure 1), which produced by Shenzhen City Boyee chemical mechanical Co., Ltd. in China. The final dispersion concentration was 3 wt % MWCNTs that remained homogeneous when stored for >2 months. The key processing parameters of the nano sand-mill were as follows: a linear velocity of 10.5–11.5 r/min, 0.8–1.0 mm zirconium balls of zirconium oxide as the grinding medium, 60%–70% filling amount, and cooling water temperature 5 ± 3 °C.

### 2.3. Preparation of Specimens

A mixture of cement paste containing MWCNTs was prepared for FE-SEM observation. The MWCNTs were added in the amount of 0.3% bwoc, and the water/cement ratio of the cement paste was 0.5. The cement paste was analyzed after 28 days of standard curing (RH: ≥95%, T: 20 ± 1 °C) in a standard curing box.

Five mixtures of UHSC were prepared by mixing cementitious materials (cm), aggregates, MWCNTs, water, and SP. The ratio of water-to-cementitious materials (w/cm) was maintained as 0.20 for all the concrete admixtures. MWCNTs were added at levels of 0.00%, 0.03%, 0.05%, 0.10%, and 0.15% bwoc in the UHSC mixtures. The UHSC samples were denoted as CNT00, CNT03, CNT05, CNT10, and CNT15. The mixture proportions for the UHSC are shown in Table 3.

Prior to pouring into a concrete mixer, the aggregates were air dried at room temperature. Sand was poured and mixed with the cementitious materials (C, SF, and BS), followed by the addition of coarse aggregates. All the dry materials were mixed for 2 min before water and SP were added. For the mixture containing MWCNTs, the dispersion of MWCNTs was first dissolved in water. After adding water and SP, the concrete mixtures were mixed for 4 min. After pouring the mixtures into oiled molds, the specimens were compacted on a vibration table. Then, the specimens were covered with a plastic sheet for 24 h. All the specimens were cured in a standard curing room (RH: ≥95%, T: 20 ± 2 °C) until the specified testing age.

### 2.4. Test Methods

#### 2.4.1. Flexural and Compressive Strength Tests

To evaluate the effect of CNTs on the mechanical properties of the UHSC samples, flexural and compressive strength tests were conducted on cuboids of 100 mm × 100 mm × 300 mm with a loading rate of 20 kN/min and cubes of 100 mm × 100 mm × 100 mm with a loading rate of 1.0 MPa/s according to the GB/T50081-2002 standard [38], respectively. Each test was conducted in triplicates. The flexural strength was calculated using the following formula:

(1) $$f=\frac{3FL}{2bh^{2}}$$

where f is the flexural strength (MPa), F is the failure load (N), *L* is the span between two supporting points (200 mm), and *b* and *h* are the width and height of the specimens (mm), respectively.

#### 2.4.2. Accelerated Chloride Permeability Tests

The conventional permeability test methods (e.g., water permeability or gas permeability test) cannot be applied to high performance concrete because it is very dense and impermeable [39]. Because the UHSC developed in this study had an extremely low permeability, the rapid chloride permeability test method, NEL method, [40] was used in this study.

**Figure 2:**
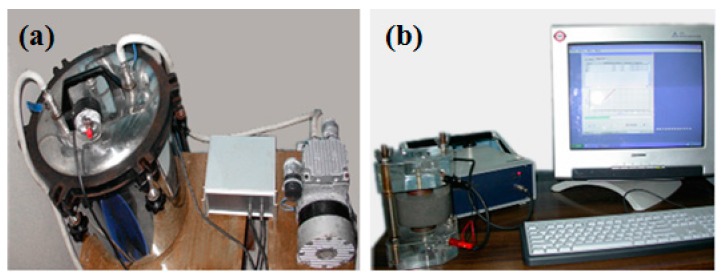
Instrument used in the chloride permeability test. (**a**) Brine saturation device under vacuum; (**b**) test equipment.

The specimen size for the chloride permeability test was Φ 100 mm × 50 mm, and each test was conducted in triplicates. The specimens were washed thoroughly and then placed in a vacuum box as shown in Figure 2a to expel all the air from the internal voids. After the vacuum suction was maintained for 6 h at a vacuum level of 0.06 atm, a saturated NaCl solution (4 M) was injected into the vacuum box, and the specimens were submerged in brine for 4 h at 0.06 atm. Then, the vacuum pump and valves were closed, and the specimens were soaked for another 18 h, thus filling all the voids with brine. Finally, the specimens were removed and sandwiched between two copper electrodes under an 8 V low voltage, as shown in Figure 2b [41].

#### 2.4.3. Morphology Observation

The field-emission transmission electron microscopy (FE-TEM) micrographs of the MWCNT samples were obtained using a PHILIPS TECNAI 10 instrument (Amsterdam, The Netherlands) working at 100 kV.

The cement paste sample with a size of approximately 5 mm × 5 mm × 5 mm was prepared at 28 days after being crushed. The sample was kept in alcohol till the SEM observation. The field-emission scanning electron microscopy (FE-SEM) micrographs of the cement paste samples were recorded using a Nova NANOSEM 430 instrument (FEI, Hillsboro, OR, USA) working at 10.0 kV.

## 3. Results and Discussion

### 3.1. Mechanical Properties

**Figure 3:**
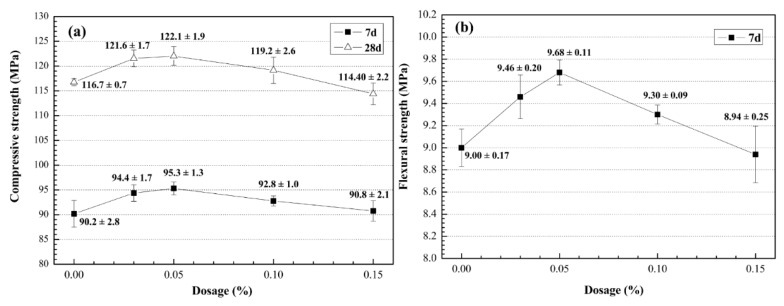
Mechanical properties of UHSC containing MWCNTs. (**a**) compressive strength; (**b**) flexural strength.

The results of the strength tests are shown in Figure 3. The compressive strength of the specimens was higher than 114.40 MPa at 28 days. Because the compressive strength was measured using nonstandard samples, the test results were multiplied with a strength conversion coefficient of 0.95 [36]. Apparently, the compressive strength of the concrete specimens prepared in this study reached the technical requirement of UHSC [36,37]. The compressive and flexural strengths increased with the percentage of MWCNTs until they reached 0.05% bwoc. Then, the strengths decreased with increasing percentage of MWCNTs from 0.05% to 0.15% bwoc. As shown in Figure 3, the specimens containing 0.03% and 0.05% MWCNTs bwoc exhibited 4.66% and 5.65% increase in compressive strength at 7 days; 4.20% and 4.63% increase in compressive strength at 28 days; simultaneously 5.11% and 7.56% increase in flexural strength at 7 days than the control UHSC sample, respectively. Clearly, the best result was obtained for 0.05% MWCNTs bwoc. In the literature, it has been concluded that small amounts of MWCNTs of approximately 0.02–0.10 wt % provide the best improvements in the mechanical properties of cement-based materials [26].

**Figure 4:**
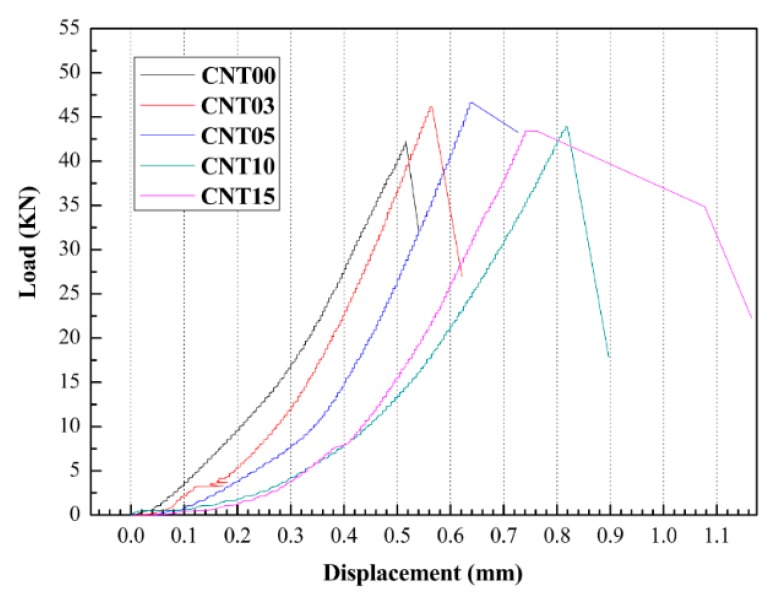
Load-displacement curves of UHSC incorporating MWCNTs. CNT00, CNT03 CNT05, CNT10, and CNT15 denotes the UHSC sample containing 0.00%, 0.03%, 0.05%, 0.10%, and 0.15% MWCNTs bwoc, respectively.

Figure 4 shows the typical flexural stress–strain curves of the UHSC containing MWCNTs. The addition of MWCNTs significantly increased the deformation ability of UHSC. The failure displacement increased with increasing MWCNT content. Similar results were obtained in the literature [23,29].

### 3.2. Chloride Permeability

**Figure 5:**
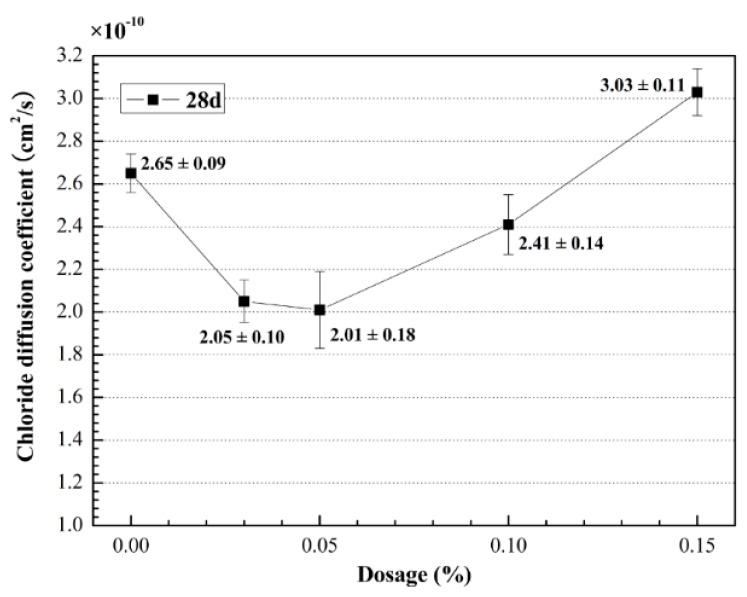
The chloride diffusion coefficient of UHSC with different content of MWCNTs.

The results of the chloride permeability tests are shown in Figure 5. The effectiveness of MWCNTs in increasing the resistance to the chloride penetration of UHSC increased in the order CNT05 > CNT03 > CNT10 > CNT00 > CNT15, consistent with the variation between the strengths and percentage of MWCNTs (Figure 3). As shown in Figure 5, the chloride diffusion coefficient of CNT03, CNT05, and CNT10 samples decreased by 22.8%, 24.0%, and 8.8% compared to that of CNT00, respectively. These results indicate that the addition of MWCNTs significantly affected the chloride permeability of UHSC.

In the literature, previous studies concluded that the increase or decrease in the resistance to chloride penetration is essentially because of the improvement or degeneration of the pore structures of concrete [41]. The fact that the addition of MWCNTs improved the resistance to chloride penetration of UHSC indicates that the porosity of UHSC decreased, and the pore sizes decreased because of the addition of MWCNTs [29]. However, the chloride diffusion coefficient of UHSC showed an increasing trend when the percentage of MWCNTs was higher than 0.05 wt %. These results can be attributed to the addition of surfactant PVP. The presence of PVP caused foaming inside the cement matrix during the mixing, consistent with that reported in the literature [42]. In this study, the mass ratio of PVP to MWCNTs was 2:1. With increasing percentage of MWCNTs from 0.10% to 0.15% bwoc, a lot of foam was generated by PVP for MWCNTs to improve the pore structure of UHSC, and the use of a defoamer was not sufficient to eliminate the effect of foam completely. This is one of the main reasons why the strength of UHSC did not improve continuously with increasing content of MWCNTs.

### 3.3. Micrograph

To successfully apply MWCNTs for the reinforcement of cement matrix, two key requirements must be satisfied: good dispersion and optimal bond strength [43].

**Figure 6:**
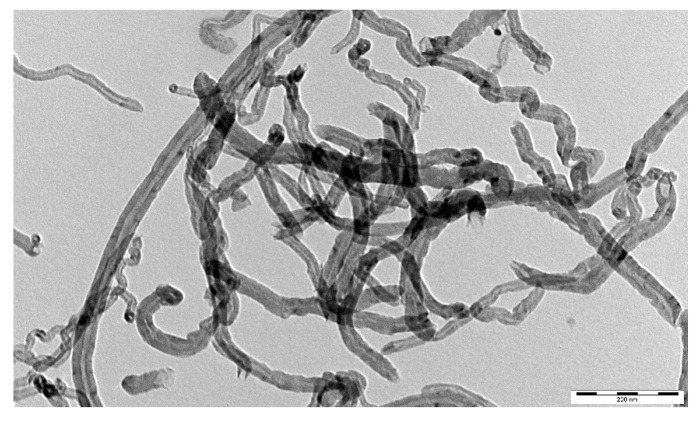
TEM image of MWCNTs. Bar = 200 nm.

**Figure 7:**
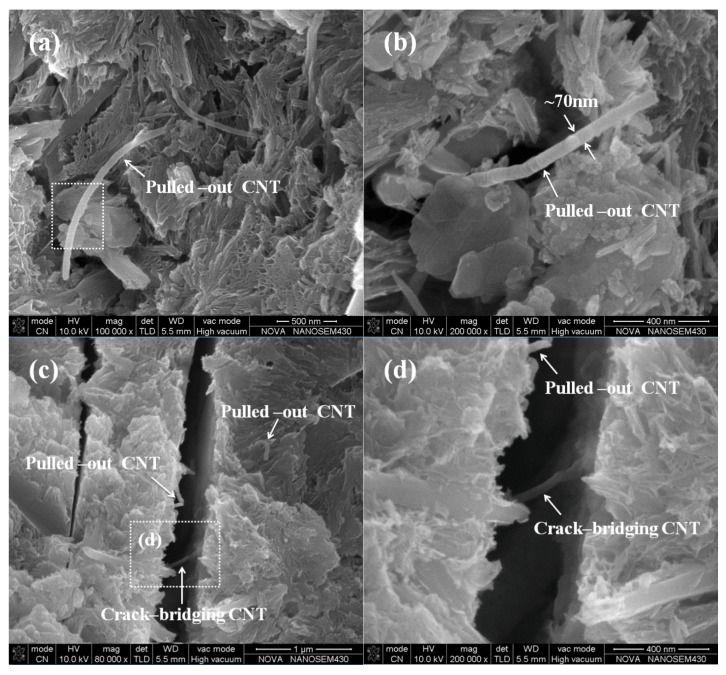
SEM images of pulled-out and crack bridging MWCNTs in cement matrix: (**a**) A typically pulled-out CNT with the cement hydration attaching on the surface; (**b**) a pulled-out CNT with about 70 nm in diameter; (**c**) pulling out and crack bridging of CNTs; (**d**) local amplification for the crack-bridging CNT in (**c**).

Figure 6 shows a micrograph of the original MWCNTs. The MWCNTs were typically entangled hollow tubes with 20–40 nm outer diameters. Figure 7 shows the distribution of the MWCNTs in a cement matrix. Clearly, the MWCNTs were well dispersed in the cement matrix with each MWCNT existing individually, and no MWCNT aggregate was observed in the matrix. Therefore, the first requirement was achieved.

Figure 7a,b show the pulled-out MWCNTs on the fracture surface of the hardened cement paste. Figure 7a shows that a small amount of cement hydration products attached to the surface of the pulled-out end. Furthermore, Figure 7b shows that a pulled-out MWCNT with ~70 nm diameter was larger than that of the original MWCNTs (20–40 nm), indicating that the pulled-out MWCNT was tightly wrapped with the hydration products. However, a large amount of cement hydration products attached to the surface of MWCNTs modified with a H_2_SO_4_ and HNO_3_ mixture (covalent treatment) [23]. The smooth surface of the pulled-out MWCNT corresponds to the nanotube-matrix interface shear failure, whereas the MWCNT surface covered with the hydration product corresponds to the matrix failure. The matrix failure indicates that the bonding strength between the MWCNTs and cement matrix is strong [30]. Thus, compared to covalent dispersion, the noncovalent dispersion maintains the superior properties of MWCNTs, but does not provide high bond strength between the MWCNTs and matrix [44]. This may be an important reason for the limited increase in strengths of UHSC mentioned in Section 3.1.

Notably, an individual MWCNT that acts as a bridge across a crack was observed except for several pulled-out MWCNTs (Figure 7c). If this crack-bridging CNT is observed at a larger amplification (Figure 7d), clearly the two ends of the MWCNT were embedded in both sides of the crack with ~0.4 µm in width. In comparison, microfibers generally require a few micrometers of crack opening for developing a noticeable crack bridging stress [30]. These results show that MWCNTs act as bridges across microcracks that cannot be achieved by conventional microfibers and therefore guarantee the load transfer under tension [23], which is helpful to improve the deformation ability and durability.

## 4. Conclusions

The following conclusions have, hence, been drawn:

1. A new method proposed for dispersing MWCNTs in water is effective. The FE-SEM observation shows that the MWCNTs were well dispersed, and no MWCNT agglomerate was visible in the cement matrix.
2. The mechanical properties increased in the presence of the optimum percentage of MWCNTs (0.05% bwoc). Particularly, the compressive strength of UHSC containing 0.05 wt % MWCNTs at 28 days increased by 4.63% than that of UHSC without MWCNTs (116.7 MPa). Moreover, the addition of MWCNTs significantly increased the flexural strength and deformation ability of UHSC.
3. Compared to the relatively small increase in strengths, a significant improvement was observed in the resistance to chloride penetration by incorporating MWCNTs. The chloride diffusion coefficient of UHSC containing 0.05 % MWCNTs bwoc decreased by 24.0%.
4. The morphology of the hardened cement paste was observed by FE-SEM analysis to understand the mechanism of improvement with MWCNTs. The microstructural studies indicate that MWCNTs act as bridges across microcracks, thus guaranteeing the transfer of the tension load. Furthermore, a lot of MWCNTs were pulled out from the matrix without rupture failure.
5. The application of MWCNTs in UHSC needs further study, and covalent treatment should be considered for dispersing MWCNTs.

## Figures and Tables

**Table 1:** Material properties of multi-walled carbon nanotubes (MWCNTs).

Diameter (nm)	Length (μm)	Purity (wt %)	Ash (wt %)	Specific Surface Area (m^2^/g)
20–40	5–15	>97	<3	80–140

**Table 2:** Chemical composition and physical properties of C, SF, and BS. LOI, SG, and SSA are the abbreviation of loss on ignition, specific gravity, and specific surface area, respectively.

Material	Chemical Composition (wt %)							Physical Properties	
	SiO_2_	Al_2_O_3_	Fe_2_O_3_	CaO	MgO	SO_3_	LOI	SG	SSA (m^2^/kg)
C	20.13	4.53	4.11	63.88	1.35	2.28	2.82	3.10	331
SF	93.85	0.69	0.17	0.75	1.22	0.41	1.88	2.20	~20,000
BS	44.91	14.86	–	31.08	7.18	0.65	1.80	2.83	1228

**Table 3:** Mixture proportions of ultra high strength concrete (UHSC) incorporating MWCNTs.

NO.	w/cm	CNTs (wt %)	TP (vol %)	Quantities (kg/m^3^)							
				C	SF	BS	CNTs	FA	CA	W	PCs
CNT00	0.2	0.00	0.13	420	60	120	0	798	976	120	15
CNT03	0.2	0.03	0.13	420	60	120	0.126	798	976	120	15
CNT05	0.2	0.05	0.13	420	60	120	0.21	798	976	120	15
CNT10	0.2	0.10	0.13	420	60	120	0.42	798	976	120	15
CNT15	0.2	0.15	0.13	420	60	120	0.63	798	976	120	15
